# Stakeholder valuation of soil ecosystem services from New Zealand’s planted forests

**DOI:** 10.1371/journal.pone.0221291

**Published:** 2019-08-22

**Authors:** Graham Coker, Mathis Richard, Karen Bayne, Simeon Smaill, Loretta Garrett, Amanda Matson, Steven Wakelin

**Affiliations:** 1 Forest Systems, Scion, Riccarton, New Zealand; 2 AroSup Dijon, Dijon, France; 3 Forest Systems, Scion, Rotorua, New Zealand; 4 BioProtection Research Centre, Lincoln University, Lincoln, New Zealand; Murcia University, SPAIN

## Abstract

The goal of this study was to determine if there were differences among stakeholders in the values they attribute to soil ecosystem services from plantation forests in New Zealand. Groups of forest-associated stakeholders were identified (e.g. land owners, forest owners, wood processors, and recreational forest users) and surveyed to assess their cultural background (indigenous New Zealand Māori or not) and then the relative importance they placed on 10 forest soil ecosystem services. Across all survey respondents, very high importance was placed on the ability of soils to sustain forest growth across multiple plantings/rotations (sustainable production). Interestingly, this was more highly valued than maximising short-term production. Māori placed greater importance on forest ecosystem resilience, provenance and kaitiakitanga (*sensu* stewardship of resources), water quality, and harvest of food and/or medicines from forests than non-Māori. These results demonstrate inherent cultural differences in valuing the range of forest ecosystem services that soils support. It is important that cultural views are understood and integrated into future soil health testing schemes to reflect the needs of all stakeholders. Ultimately, this work will help increase the sustainability of planted forest ecosystems in New Zealand, ensure the forestry sectors social licence to operate, and add value to forest products by demonstrating environmental and cultural stewardship of forest products.

## Introduction

The productivity and sustainability of both native and managed terrestrial ecosystems are underpinned by effectively functioning soils [1]. Not only do soils influence above and below ground soil productivity, they drive energy flows through decomposition, moderate the cycling of nutrients, impact environmental quality (e.g. water and air quality, erosion) [2] and constitute one of the largest reservoirs of biodiversity globally [3,4]. Not surprisingly, there is considerable interest in maintaining soil health with a view towards protecting natural capital [5], particularly as ecosystems are increasingly subject to anthropogenic influences, ranging from extreme climate events to land use change [6], and human influences on land management regimes.

While there have been many attempts to define soil health [7], one of the most widely used is that of Doran and Safley (1997) [8]: “*the continued capacity to function as a vital living system*, *within ecosystem and land use boundaries*, *sustain biological productivity*, *to promote the quality of air and water environments*, *and maintain plant*, *animal*, *and human health”*. This definition not only recognises the importance of multi-functionality, but the inherent ‘vitality’ of soil; i.e. a complex ecosystem in which processes that support primary production and where ecosystem functions are expression of the biology of the ecosystem and its interactions with the physical, chemical, and plant-based (roots, litter) components of the environment [9,10]. The underlying importance of soil biological diversity and activity in wider ecosystem functioning is driving international efforts to integrate various biology-based metrics into soil health testing [11–14].

Under New Zealand’s Resources Management Act (RMA), regional councils have legislated responsibility for assessing the potential detrimental effect(s) of human activities on environmental quality [15]. However, the RMA does not specify which soil properties should be monitored. In order to unify soil-based health reporting, 500 soils across New Zealand are monitored for a suite of physicochemical attributes including acidity (pH), total carbon, total nitrogen, Olsen phosphorus, bulk density, and macroporosity [16]. To date, however, there has been no inclusion of biological parameters in such monitoring. This is notable, particularly given the potential gains from managing soil biological resources in countries such as New Zealand [17]. As New Zealand is highly reliant on primary production for economic growth, while also rapidly trying to mitigate environmental impacts of productive land use for a sustainable future, this is of increasing importance.

Historically, there have been a number of issues limiting the application of biological attributes into soil health assessment criteria used for national scale monitoring [11]. These include cost, scalability (throughput), reproducibility, sensitivity/responsiveness to purpose of application (e.g. land use change), technological readiness, ease-of-use, policy readiness, standardisation, and infrastructure availability [11, 12]. Perhaps most importantly, however, are issues of ‘meaningfulness’, and ‘understandability’ by the end-users [18]. For example, variation in microbial community composition can be a sensitive indicator of land use change [19]. However, unless changes in the community are important (e.g. achieving a specific goal related to preserving natural biological diversity), there is little functional context; i.e. what does the datum/statistic mean, and how should the results be interpreted in terms of soil health?

It has not been until relatively recently that soil microbial communities, their intrinsic biological diversity, and role in delivery of a range of ecosystem functions, have been considered within an ecosystems services framework [5]. In the context of this study, this is the range of benefits to humans that is realised by an appropriately-functioning forest soil ecosystem. This encompasses the role of soils in tree growth (provisioning ecosystem services; wood and fibre), as a filter and regulator of water flow (regulating ecosystem service), through to supporting ecosystem services (food and game, biodiversity). The importance of each of these can further be assessed within a cultural context. With perspective towards benefits to humans and society, this framework has focused on the importance of soil biology as a critical component of terrestrial ecosystems [5]. Furthermore, it is logical that anthropogenic stresses, whether expressed immediately by such events as metal contamination or long-term through climate disruption, may have impacts on soil biology and the ecosystem services they support. Accordingly, the framework provides an opportunity to manage trade-offs and seek balance between human manipulation of ecosystems (e.g. to increase productivity) and provision of other ecosystem services such as clean water.

Soil health testing and monitoring is being adopted or proposed in the E.U., USA, and many other jurisdictions [7,12,20,21]. These monitoring schemes are being driven, in part, by consumer sentiment that provision of goods meet societal expectations for environmental best practice. Indeed, the demonstration of environmental stewardship through the use of accredited sustainability labelling of products is increasingly seen as a value-added proposition [22]. Labelling informs consumer choice selection among products [23], and can help to maintain the social license to operate for various productive sectors [24]. International conventions cover environmental performance of land use [25], and provisions for environmental benchmarks are also increasingly an important component of bi- and multi-lateral trade deals [26]. Via consumer pressure, societal awareness, application of regulatory or policy tools, and/or by the forestry sector embodying ‘environmental stewardship’ as a value for forest-based products, the ability to measure and report soil health values for food and fibre production systems is becoming increasingly important.

There are many hundreds of individual biological endpoints that could potentially be integrated into soil health monitoring schemes [27]. Examples are measures of microbial diversity, presence of keystone or indicator taxa, soil functions such as nutrient cycling and decomposition, nitrogen fixation, resilience of the soil ecosystem to disturbance, and many others [27]. However, these vary widely in attributes such as scalability, reproducibly, interpretability, throughput, cost, and so forth [28]. As such, the selection of indicators for effective soil monitoring is challenging, and requires a well-structured approach [13]. Ultimately, however, the indicators selected must inform the application to which they are targeted; i.e. is the monitoring scheme focused on comparing land-uses, or achieving sector-specific goals within a single land use? Furthermore, to inform monitoring or management schemes, panels of indicators must closely align with ecosystem services or functions of value. These values are of primary concern, as they will directly affect the efficacy of soil biological indicators to inform land management decisions for desired outcomes. In the context of this work we are explicitly focusing within planted forest ecosystems (i.e. not between planted forests and other land-use types). Accordingly, this allows us to be more targeted in the future development of indicators that provide the most benefit to sustaining forest soil ecosystem services. The key question is, what forest soil ecosystem services are of most importance to different stakeholders?

### Context of planted forestry in New Zealand

Planted (commercial) forestry is an important land use in New Zealand. Planted forests cover 1.7 m Ha—or approximately 9% of New Zealand’s land area that is not held in the conservation estate—and earns NZ$4.8 billion export dollars annually [29]. The forestry sector is particularly important to regional economies, who not only rely on forests for employment, but as places for recreation and tourism (mountain biking, walking, hunting etc) and a source of clean water [30]. In many areas, planted forests are interspersed with other land uses, both native and productive, and the impacts of forestry on these are important. These include, for example, regulation of catchment-level water flow and stabilisation of hill slope soils from erosion, through to offsetting greenhouse gas emissions from livestock-based agriculture (particularly methane from ruminants) [31]. Thus, planted forests, in New Zealand and elsewhere, have much greater value to society than their economic contribution alone [30].

The planted forest sector in New Zealand is relatively unique. Approximately 90% of the forestry estate is a single species, *Pinus radiata* D. Don, and these are grown in stands of uniform age and density. With an average rotation length of 28 years, these are regarded as highly productive, short-rotation forestry systems [1]. While there are significant management and silvicultural benefits to having uniformity in planted forests (single species and age class), there are also risks associated with potential pests and diseases, and questions as to whether forests can sustainably deliver a myriad of ecosystem services [32, 33]. As a signatory to the Montréal Process [34], New Zealand is obligated to monitor and report on the impact of management on the maintenance of ecosystem health and vitality in these forests, including the soil biological components associated with fundamental ecological processes (Criterion Three of the Montréal Process). However, a lack of clarity around appropriate biological indicators for soil has limited meaningful reporting in this area [35].

In addition to providing ecosystem services for the country as a whole, planted forests in New Zealand also have a strong cultural value for Māori [36]. These values may or may not differ in importance from those of the wider community and, as such, need to be formally considered when valuing ecosystem services. By formally accepting and integrating indigenous knowledge and values into soil health monitoring, we will be better positioned to inform land use decisions that affect indigenous and /or local communities [37]. Of particular relevance is the Māori cultural connection to the land (Papatūānuku; mother earth which supports and nourishes life) and, extending from this, the importance of stewardship (kaitiakitanga; broader sense extending to nurturing and caring) to protect the vitality of soils and the ecosystem they support [38]. Given the fundamental connection of Māori culture with soil ecosystems, we hypothesise that values associated with ecosystem sustainability and protection of soils, i.e. as a vital (living) ecosystem, may be stronger than those held by the wider community.

In order to align biological indicators with ecosystem services of importance to planted forest soils, we undertook a survey of a range of forestry stakeholders. The aims of this survey were to rank the importance of forest soil ecosystem services to different planted forest stakeholders, and to identify differences between Māori and non-Māori that may require integration into a future assessment programme.

## Methods

A web-based survey system (SurveyMonkey) was used to query a range of forest stakeholders about the importance (values) they place across a range of forest ecosystem services. The survey assured anonymity, with only primary metadata collected related to the participant’s relationship with planted forests, geographic region in New Zealand, etc. To encourage the voluntary provision of personal contact data, which was necessary to communicate feedback and results to survey participants, a reward-based system was instituted. The survey questions are provided as supplementary information (S1 Table).

The following seven groups were used as classifiers, being key ‘stakeholders’ in the ecosystem services that forests soils support: forest owners, forest managers, land owners, land managers, wood processors, recreational forest users (fishing, hunting, etc), or ‘others’ with a vested interest in forest soils. Community metadata was collected to provide a better understanding of motivations for participant responses, and was not formally included in the analysis. After identifying the 7 stakeholder groups, we used a combination of targeted and general approaches to seek additional participation. Invitations to participate in the survey spanned communication on various social media platforms, through to use of special-interest distribution lists (e.g. wood processors communication list). Respondents were asked if they identified as Māori, and/or represented a Māori community, Māori trust, or other such organisation. This information allowed the identification of responses coming from a Māori cultural perspective.

A range of ecosystem services or ecosystem functions that are supported by forest soils were broadly identified from those presented in the UN’s Millennium Ecosystem Assessment framework [5]. The survey queried how important 10 forest soil ecosystem services were, on a 0–100 scale, for each participant. The ecosystem services, presented in a randomised order, were:

- Maximising production of the current forest rotation;
- Achieving sustainable production (i.e. over multiple rotations);
- Preserving soil biodiversity;
- Controlling pests and diseases;
- Forest ecosystems resilient to significant disturbance events (e.g. climate impacts);
- Provision of non-wood forest products such as harvest of foods and medicines;
- Provision of drinkable water from forest streams;
- Provenance and/or kaitiakitanga (protection and guardianship) of forest soils;
- Storing soil carbon;
- Other values not listed above.

The results for the scale data (0–100) were initially analysed following a multivariate approach. Similarity in response (co-variance) to answers was conducted using cophenetic correlation (Pearson’s method) with type-3 similarity profile analysis (SIMPROF) testing and permutation-derived p-values [39]. In this method, similarities in response patterns of each of the ecosystem services was determined across the stakeholders (index of association), and the statistical reliability of groups (clusters) tested using type 3 SIMPROF tests [39]. This allowed the significantly-similar groups of ecosystem services to be identified; i.e. those with variable sets that co-varied in their response patterns across the various stakeholders. Variation in overall response patterns was interpreted using metric multidimensional scaling (mMDS) based on Euclidean distances calculated among the samples. Bootstrapping was used to determine confidence of groupings (i.e. based on stakeholder association). Formal testing of variation in responses among stakeholders and between Māori and non-Māori communities was conducted using permutational multivariate analysis of variation (PERMANOVA; [40]). For treatment effects where differences were evident (Māori v. non-Māori responses) similarity percentage (SIMPER; [41]) testing was used to characterise the main response variables contributing towards these differences.

Given the potential for survey participants to give all the ecosystem services (see previous section) a similarly high or low level of importance (e.g. multiple ranked at 100% importance), a subsequent question was used to force selection of the three most important soil ecosystem services. The top three responses provided by forced ranking were weighted, with the most important (ranked 1^st^) given a value of 3, second 2, and third most important 1. The results were compared for Māori and non-Māori groups, and then across the range of forest stakeholders. No ‘Māori × stakeholder interaction’ analysis was conducted due to insufficient replication at this level. The data did not pass tests for normality and, as such, were analysed using non-parametric ANOVA with permutation-based testing of effect significance (PERMANOVA). Wilcoxon signed-rank test (2 sample testing implemented in R [42]), a non-parametric alternative to Students t-test, was then used to determine if the weightings for pair-wise sets of forest soil ecosystem services differed between Māori and non-Māori. To assess differences in views among the wider stakeholders, ordination by principle components analysis (PCA) was conducted. Vectors associated with each of the ecosystem services linked (based on SIMPER pair-wise testing [41]) to separation across the ordination were overlaid on the plot.

Research was conducted with knowledge and approval of the technical steering committee for the research program that funded this work. Similarly, work-plans were approved by both research institutes involved; Agrosup Dijon and Scion. Given voluntary participation, anonymity of responses, and full disclose that the information would be used for publication, further formal review by an ethics committee was not sought.

## Results

The survey attracted 169 respondents; of these, 145 provided completed responses suitable for analysis. A very small number of respondents (n = 2) did not provide information on their stakeholder association; these were included under ‘others’. Importantly, respondents identifying as Māori, or representing Māori community interests, accounted for 9.7% of the overall survey returns. The breakdown of survey returns by sector (community of interest), years of experience, and primary geographic region of engagement with planted forests in New Zealand, is given in the Supporting Information (S1 Appendix).

### Patterns of stakeholder responses to valuing forest soil ecosystem services

Similarity in overall patterns in valuation (0–100 scale for each) of ecosystem services were compared across stakeholders, between respondents identifying as Māori or non-Māori, but not at a stakeholder x Māori level. Overall testing of the multivariate data was conducted using a multivariate equivalent of ANOVA, with permutation of the underlying data to create a distribution of expected outcomes against which likelihood outcome of the result statistic could be assessed (i.e. PERMANOVA); as such, the results in Table 1 can be interpreted in an analogous way to a traditional ANOVA summary table.

**Table 1:** Summary PERMANOVA testing for similarity in responses, among stakeholders and Māori, for overall similarity in responses to the quantification of importance of 10 soil ecosystem services in planted forest ecosystems.

Source	Pseudo F	p_(perm)_^a^	√CV^b^
Stakeholder^c^	1.882	0.006	13.80
Māori v other	7.908	0.001	35.02
Residual			64.77

^a^ p_(perm)_ is the p-value derived from permutation (999 times) based testing.

^b^ √CV = is the square-root of the component of variation, a measure of the effect size for each component in the analysis.

^c^ Stakeholder = forest owners, wood producers etc. See full description in the main text.

Testing of the main treatments found both stakeholder (p = 0.006) and Māori association (p = 0.001) had highly significant differences in overall response patterns amongst their respective groups (Table 1). Inspection of the partitioning of components of variation (√CV) between these main treatments (Table 1) showed that Māori-association was much stronger than stakeholder. However, as stated before, both are statistically significant.

SIMPER testing was used to determine the ecosystem services in which differences in values contributed to the overall Māori and non-Māori group difference; these are summarised in the S1 Table. The analysis found Māori to more likely to place higher value on sustainable harvest over rotations, provenance and kaitiakitanga, maximising production, carbon storage, disease suppression, and maintenance of biodiversity (S1 Table).

To investigate ecosystem values that contributed towards overall differences in responses among stakeholders, pair-wise comparisons (PERMANOVA; as before) among the groups was conducted. Significant differences were present when responses from the recreational users group were compared with forest owners (p = 0.002), others (p = 0.033), land owners (p = 0.017), and forest managers (p = 0.001). The views among all other stakeholders were similar (p>0.05), the exception being forest owners and forest managers (p = 0.01). The results are summarised in an ordination plot (PCA; S2 Fig). In this plot, the separation (degree of difference in overall valuation of the ecosystem services) among the 7 stakeholders is overlaid with variables (i.e. the individual ecosystem services) that are most high ranked with explaining variation among each of the pairs of significantly different groups. For example, SIMPER testing found differences between forest owners and forest managers were best explained by values of ‘provenance and kaitiakitanga’, and ‘sustainability in harvest over multiple rotations’ (both were valued higher by forest owners than forest managers). These variables, and others associated with differences among pair-wise tests, are overlaid on the S2 Fig. The summary results show that the main axis (PCA 1) explained 35.8% of the variation across the stakeholder groups, and the second component (PCA 2) 16.6%. The importance of provenance and kaitiakitanga, as linked to delivery of forest soil ecosystem services, was strongly associated with separation in groups across the main axis. Carbon storage had a similar directional influence but of smaller magnitude (S2 Fig). Sustainable production and maximising short-term production were associated with separation in use group responses across the second PCA axis (S2 Fig).

Across all 145 survey respondents, the variation in responses (valuing forest soil ecosystem services) fell into four distinct patterns (grouping based on cophenetic distances; S3 Fig). The ability of soils to support sustainable harvest over rotations was independent from the other responses; this was seen as highly important across all respondents. The value placed on maximising production and disease protection varied in a similar manner across respondents. Soil carbon storage, maintenance of biodiversity, and resilient forest ecosystems formed a distinct response cluster (Group type C; S3 Fig). Finally, the responses for provision of clean water, harvesting food from forests, and provenance / kaitiakitanga also formed a distinct group.

### Comparing the value of forest soil ecosystem services among stakeholder groups

Results for valuations of ecosystem values across forest stakeholders and those identifying as Māori or non-Māori, are given in S4 and S5 Figs. However, there was potential for participants to value multiple ecosystem values very high or very low. As this would render these inseparable for analysis purposes, participants were also required to rank the top 3 ecosystem services by importance (i.e. forcing separation). These were analysed in both a weighted and non-weighted assessment; i.e. where the first ranked gets higher nominal value in the analysis or not. Ultimately, weighting of the data made no difference to analysis outcomes. Accordingly, only the weighted data analyses are presented.

**Fig 1:**
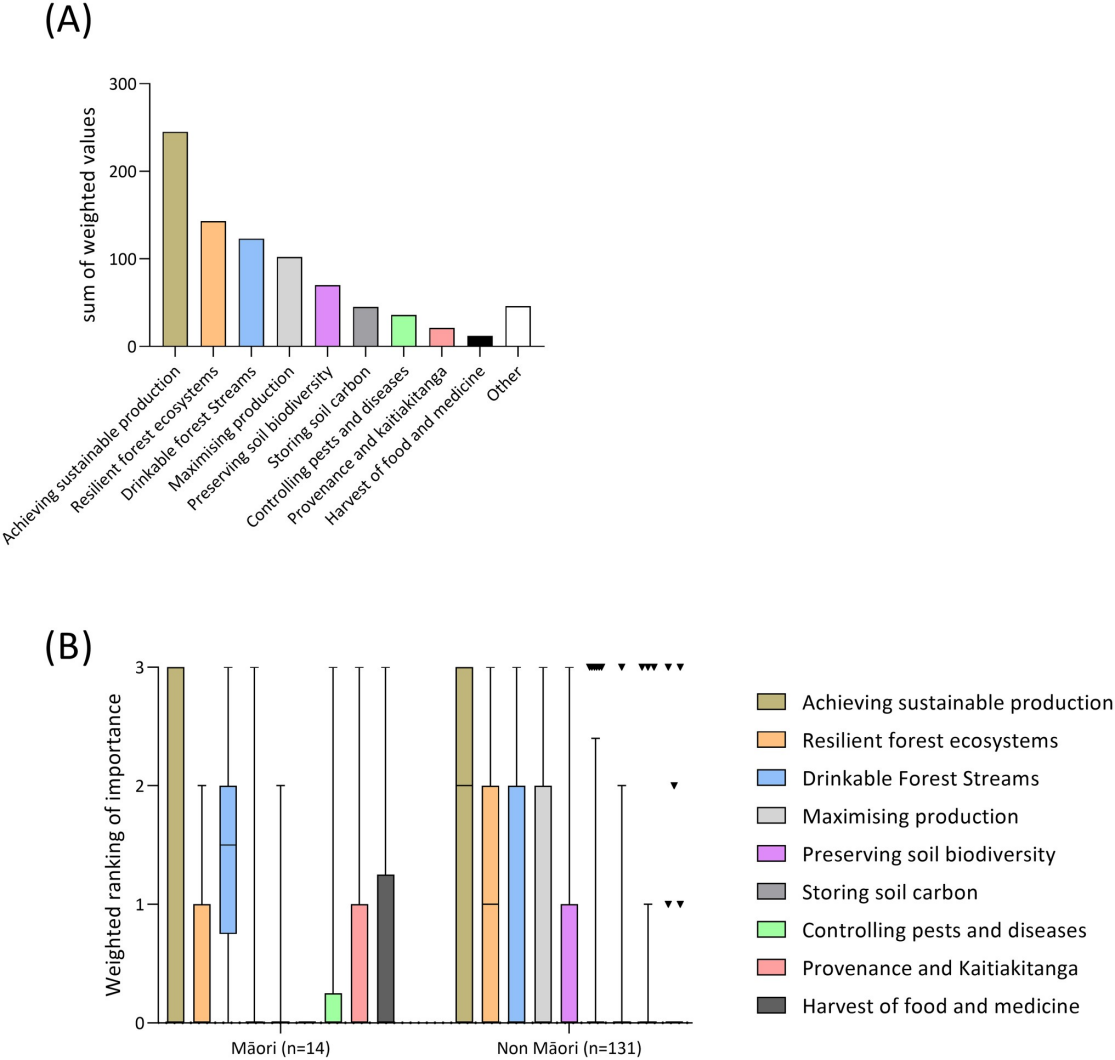
(A) Sum of top 3 ranked forest soil ecosystem services, across all stakeholder groups, and (B) separated into Māori and non-Māori survey respondents. Data were weighted such that 1^st^ ranked was scored ‘3’, 2^nd^ ranked ‘2’, and 3^rd^ ranked ‘1’. For Fig 1B, the average value and ‘whiskers’ extending from the 5–95 percentiles are given. When too few data are available to calculate the 25th to 75th percentiles (i.e. the extent of the box) a single line presenting the data/datum range is provided.

Across all stakeholders, the highest ranked ecosystem service was ‘achieving sustainable production’ (Fig 1). This was of considerably higher importance than the next most important ecosystem services, resilient forest ecosystems, drinkable forest streams, and maximising production (Fig 1A). Interestingly, these four ecosystem services were present within each of the four ‘distinct response groups’ (S3 Fig), and thus provide the highest valued indicator for each (i.e. the most highly valued representative ecosystem services for which biological endpoints may be developed).

When comparing between Māori and non-Māori, differences in average weightings were evident (Fig 1B) and the differences summarised in Table 2. Māori placed higher value on water quality (drinkable streams), provenance and kaitiakitanga, and harvest of non-timber materials (e.g. food) from forests than non-Māori (Fig 1B; Table 2). In contrast, non-Māori stakeholders placed higher average weighting on resilient forest ecosystems and sustainability of production across multiple rotations (Table 2; Fig 1B). However, both of these ecosystem services were of high importance and this was irrespective of participants identifying as Māori or not (Fig 1A).

**Table 2:** Summary Wilcoxon signed-rank testing to determine if Māori and non-Māori survey respondents differed in their ranking of importance of forest soil ecosystem services.

Forest soil ecosystem function	Wilcoxon rank	p-value
Drinkable forest streams	604	0.021
Provenance and kaitiakitanga	641	<0.001
Harvest of food and medicine	690	<0.001
Controlling pests and diseases	869	0.630
Achieving sustainable production	1072	0.277
Preserving soil biodiversity	1154	0.053
Resilient forest ecosystems	1221	0.031
Storing soil carbon	1099	0.069
Maximising production	1148	0.056

**Fig 2:**
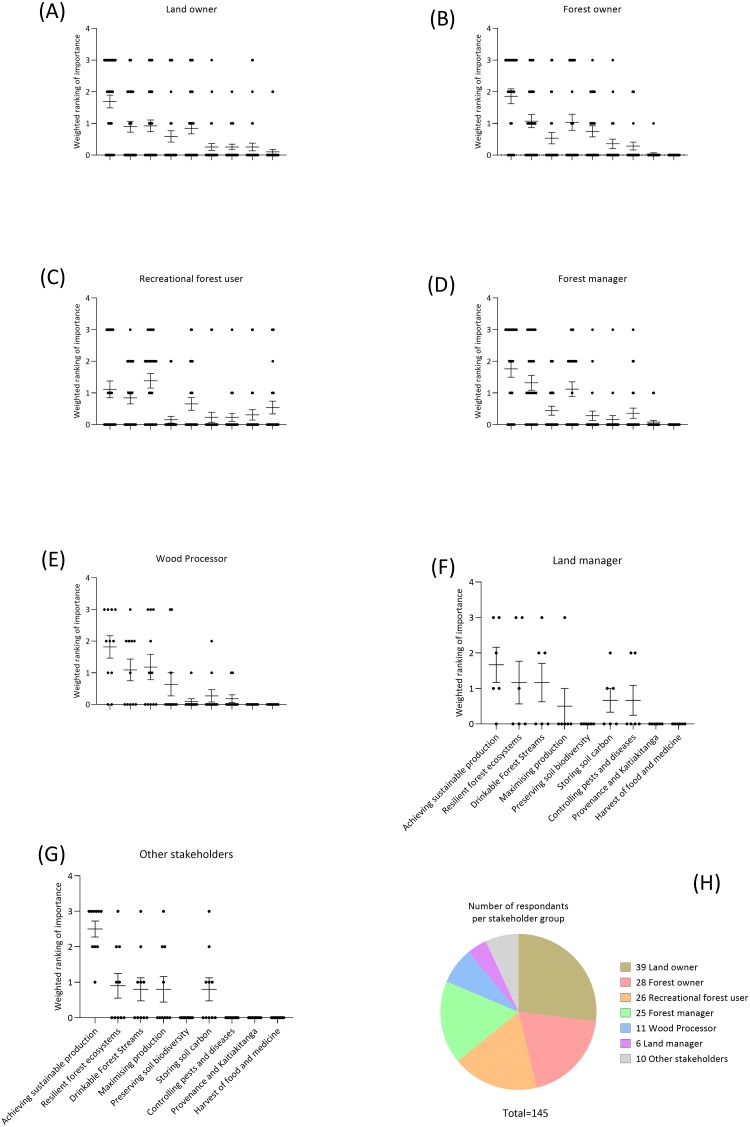
Top 3 forest soil ecosystem services as ranked by different stakeholders. Results were weighted such that the most important (1st ranked) received a value 3, 2nd ranked a value of 1, and 3rd ranked a value of 1. Numbers of respondents for each stakeholder are given in plot (h). Data are presented as scatter dot plots, with mean value on the horizontal bar and SEM on the error bars.

The data were also compared among forest stakeholders. Significant differences existed in the values placed on forest soil ecosystem services (top 3) among the groups (p = 0.005); the individual data plots are presented in Fig 2. The ability of soils to sustainably support forest production, identified as one of the most highly-ranked ecosystem services over all stakeholders (Fig 2). Stakeholders that ranked maximising production (i.e. achieving maximum, short term forest production) as of high importance were forest managers and forest owners (Fig 2B and 2D). Recreational forest users placed strong importance on provision of quality forest water as an ecosystem service (Fig 2C). The ability of soils to confer resilience to forest ecosystem function was ranked relatively high by all forest stakeholders (Fig 2) except for ‘other’ users.

## Discussion

There are a number of strong drivers for the integration of biologically-explicit indicators (endpoints) into systems for the assessment and monitoring of forest soil health. These include consumer demand, international trade agreements and maintaining social licence to operate (environmental stewardship), through to understanding ecosystem sustainability and productivity opportunities/trade-offs. Currently in New Zealand, environmental quality (including soil) is monitored by Regional Councils (RMA; [15]), however this monitoring does not include soil biological endpoints. Furthermore, monitoring within a framework based on ‘assessing the potential detrimental effect(s) of land use activities’, misses the opportunity to ‘capture the potential benefits of monitoring to increase the value, acceptance, and sustainability’ of a land use as articulated in the RMA [15]. With a focus on New Zealand’s planted forest sector, we are undertaking a structured approach towards realising the opportunities for integrating soil biological indicators into soil health monitoring. Given that there are domestic and international drivers to conduct such monitoring, this is an opportunity for the forestry sector to proactively integrate a set of indicators that address monitoring of detrimental changes in soil quality, while also supplying information regarding other valued ecosystem services. To determine an appropriate set of indicators, it is necessary to understand which soil ecosystem services are most valued, and if these values differ among stakeholders.

Across all respondents, four cophenetically-similar groups were resolved (S3 Fig): (A) sustainable production across rotations; (B) maximising production and disease protection; (C) increased ecosystem resilience to disturbance, carbon storage, and maintaining biodiversity; and (D) provision of clean water, harvest of non-wood products from the forest (e.g. hunting), and provenance and/or kaitiakitanga of forest products. These groups provide some insights into the sharing of values among the survey respondents.

Group A was represented by a single soil ecosystem service: sustainable production across rotations. This ecosystem service was also shown to be the most highly ranked in importance (Fig 1A), well ahead of maximising short-term forest production. Therefore, as most stakeholders value the ability of soil ecosystems to support sustainable, long-term production, the inclusion of soil biological indices specifically targeting this ecosystem service are evidently essential. Groups B and C may have also been a reflection of short-term vs. long-term values. Group B included productivity and disease suppression, both of which could be current-rotation issues, while Group C included biodiversity, resilience and carbon sequestration, which address long-term issues such as climate change. Interestingly, when looking at the top-ranked ecosystem services overall (Fig 1), a long-term value (i.e. resilient forests) was ranked 2^nd^ overall.

When asked to quantify the importance of forest soil ecosystem services, the response of the different stakeholders were generally similar (Fig 2), with the exception of the recreational forest users, who most strongly associated with the ecosystem services in Group D. Recreational forest users, perhaps unsurprisingly, highly valued non-wood-based services: the provision of clean water, the harvest of food and other (non-timber) products from forests, and provenance and/or kaitiakitanga (Fig 2). Water quality is a significant problem in New Zealand [43] and was, in fact, the third-most valued ecosystem service (Fig 1). Furthermore, as there is increasing demand for food and fibre products that have been grown using holistic best management practices [20], so this was also an unsurprising choice by recreational forest users.

We hypothesised that protection and guardianship of planted forest soils would mainly be of significant cultural importance for Māori [36]. The high ranking of recreational forest users in this category led to further exploration of their cultural association; this stakeholder group comprised 19% of people identifying as Māori, approximately double the percentage for overall respondents. As such, it is probable that the importance of provenance and kaitiakitanga for these stakeholders was influenced by the proportionality of Māori-cultural values held in this group. However, given the relatively low total response rate for Māori, these results need to be interpreted with due caution, and further investigation of these findings are warranted.

Comparing across all respondents, Māori respondents ranked values associated with water quality, provenance and kaitiakitanga, resilient forest ecosystems, and harvest of food and/or medicines more highly than non-Māori (all p<0.05; Table 2). These findings highlight the importance of formally integrating assessment for cultural differences in such surveys. As such, values of specific importance to Māori would be masked, as a small overall percentage, by those of the wider respondents.

Māori have close connections to planted forests in New Zealand. Forests make up approximately 10% of the total asset base supporting the Māori economy, and there are projections that Māori may eventually own or control greater than 40% of New Zealand’s planted forests [38]. Therefore, this group needs to be well represented in decision-making that affects the forestry industry. The rate of participation of respondents identifying as Māori was relatively low, at 9.7% of the total. Given this, there is potential that stronger participation by Māori may affect the results and, as such, this study should be followed up by further investigations to ensure the findings are truly representative. It should be noted however that the population size of Māori in New Zealand is relatively low and this is reflected in the response rate. However, the exploration of potential cultural perspectives on the value of soil ecosystems services was important and, indeed, formed an explicit *a priori* defined area of investigation. When specific inclusion of a stakeholder group coincides with a naturally low-percentage of that group in the survey population size, response rates are expected to be relatively low. Despite this, the findings have significance toward the stated intention to determine if there is a need to more formally include cultural values in the future development of endpoints for soil health guidelines. In addition to their intrinsic importance to Māori, there is instrumental value to be gained in developing indicators for culturally-significant soil ecosystem services. This would be of benefit, for example, to the forest product value chain through realisation of increased value or protection of market access following demonstration of environmental, social, and cultural stewardship.

Having established the values (relative importance) that different stakeholder groups place on planted forest ecosystem services, we now aim to identify a broad panel of biological indicators that may be used in the assessment of ecosystem service delivery over time, space, and forest management practices. The selection of these will be cognizant of the need for a broad range indicator attributes; i.e. cost, scalability, reproducibility, ease-of-use, etc (as described before) [11, 12]. Finally, using a network of forest field trial sites across New Zealand we aim to validate these in real-world conditions, ultimately providing a rigorously assessed, fit-for-purpose biological indicators for use by forest owners, land managers, and other key stakeholders.

Engagement with stakeholders through surveys or other processes is essential to identifying the full suite of soil ecosystem services desired from New Zealand’s planted forests, and also provides valuable insights to help ensure the content of stakeholder extension programmes remains relevant [31]. Information regarding ecosystem service preferences will also be a valuable asset for generating multiple-use planted forests that provide traditional and non-market values [44], and the decision-analysis tools required to develop and implement forest management strategies that encompass these non-market values [45].

## Conclusion

The results of this work establish the foundation for the development of a suite of biological metrics for inclusion in assessment and monitoring of soil health for New Zealand’s planted forests. Importantly, the endpoints need to align with soil ecosystem services of value to a range of forest stakeholders. Based on the study findings, these should include: (1) ensuring sustainability of forest production; (2) resilience of forest ecosystems to disturbance; (3) provision of clean water; and (4), provenance and kaitiakitanga (identity and stewardship-based cultural values). Our results have implication for anthropogenic use and long-term management of forested soil environments, especially in light of current intensive production regimes, and expected increase of forestry as a component of productive land use systems in New Zealand.

## Supporting information

S1 Appendix Survey questions. List of questions and explanatory material used for surveying stakeholders for their valuation of forest soil ecosystem services.
(DOCX)

S2 Appendix Tabulated survey response data. Tables of data of responses (counts and/or values) to questions posed in the survey of forest stakeholders.
(DOCX)

S1 Table Forest soil ecosystem services that are valued differently by Māori and non-Māori stakeholders. Summary SIMPER testing of variables contributing towards the differences between Māori and non-Māori stakeholder quantification of forest soil ecosystem services. Average dissimilarity = 15.91.
(DOCX)

S1 Fig Summary of survey respondent metadata. Breakdown of survey respondents by (a) sector/community of interest, (b) years of experience, (c) type of enterprise (private v commercial operator etc), and (d) the region of forestry interest. Note for ‘region of interest’ that more than one option was available for the survey respondents.
(DOCX)

S2 Fig PCA ordination of differences among stakeholders response to values in soil ecosystem services. Principle components analysis (PCA) ordination plot showing separation in forest stakeholders’ valuation of overall forest soil ecosystem services. Lines indicate the ecosystem values (variables) associated with separation in responses in each direction; the length of the lines is proportional to their strength. Variables were selected based on ranking in SIMPER analysis conducted between pairs of stakeholder groups found to significantly differ (p<0.05).
(DOCX)

S3 Fig Grouping of stakeholder valuations of ecosystem services into similar-response clusters. Grouping of ecosystem services by similarity in response patterns (Pearson’s correlation) across the 145 participants. Data were first normalized; clustering based on the group-average method.
(DOCX)

S4 Fig Box-plot presented summary for valuation of ecosystem services across stakeholder groups. Values placed by different stakeholders (x-axis) on a range of planted forest ecosystem services. Note many were ‘valued’ at or near 100, giving a skewed perspective of the distributions. Data includes responses from both Māori and non-Māori communities. Maintaining biodiversity refers to soil biodiversity only.
(DOCX)

S5 Fig Box-plot presented summary for valuation of ecosystem services across Māori and non-Māori stakeholder groups. Values (Y-axis) placed by survey respondents identifying as Māori and non-Māori (x-axis) on a range of planted forest ecosystem services. Note many ‘valued’ at or near 100 (Y-axis), giving a skewed perspective of the distributions. Data includes responses from across all stakeholders. Maintaining biodiversity refers to soil biodiversity only.
(DOCX)
