# Supplementary material for: Stakeholder valuation of soil ecosystem services from New Zealand’s planted forests
Source: PLoS One. 2019 Aug 22;14(8):e0221291. doi: 10.1371/journal.pone.0221291 (PMC6705829; doi:10.1371/journal.pone.0221291)

**Soil health in New Zealand's planted forests**

**Introduction**

**Planted forests cover at least 1.7 million hectares of New Zealand's land surface. Given their extent, the soils under our forests have a significant role in the provision of beneficial ecosystem services to you and New Zealand. These services could include the regulation and purification of water, supporting forest growth and wood/fibre production, through to storage of carbon. As such, monitoring and assessing the health of forest soils is nationally important.**

**Soil health can mean many different things. A useful definition is "The continued capacity to function as a vital living system, within ecosystem and land use boundaries, sustain biological productivity, to promote the quality of air and water environments, and maintain plant, animal, and human health" (Doran and Safley, 1997).**

**Currently, in New Zealand, the monitoring of soil health is based on physicochemical measures such as pH or bulk density. However, these don't recognize the inherent importance of biology and biological processes in the provision or regulation of a number of soil ecosystem services.**

**A holistic approach to soil health would include measurement of key biological properties alongside the physicochemical attributes already determined. This approach may provide stronger insights into how forest soils function and identify opportunities to increase the total productive capacity of forests, achieve greater suitability of production, or better realize the various other ecosystem services.**

**Internationally, there is significant momentum to include biological attributes into soil health testing. This is being driven by consumer sentiment and their selection of food, fibre, and other products from land uses deemed sustainable. Indeed, there may be potential to hold or expand market access, or obtain additional value from forest products, if we can demonstrate stewardship, kaitiakitanga (guardianship), and provenance from New Zealand.**

**The goal of this survey is to determine the soil ecosystem services or functions that are of most importance to different end users. From this information, we will aim to identify a set of biological indicators that can be included in future soil health monitoring practices.**

**Your view is important, and we need input from a wide range of forest users. We hope you can take part in this short survey.**

**We have estimated this survey will take approximately 10 minutes to answer, and all questions are optional.**

**Participation is anonymous, but if you would like to stay informed about this project and our results, please provide your email address at the end.**

**If you would like to enter into the draw to win a gift card of $200 value please provide contact details on the last page.**

**If you have any questions about the project or the survey, please contact Mathis Richard at Mathis.Richard@scionresearch.com,**

**Steve Wakelin at Steve.A.Wakelin@scionresearch.com, or Graham Coker at Graham.Coker@scionresearch.com**

**Acknowledging and proactively working towards Māori inclusion on soil biological health measurements provides a valued view alongside other interest groups. Do you identify as Māori, represent a Māori community, Māori trust, or other such organisation?**


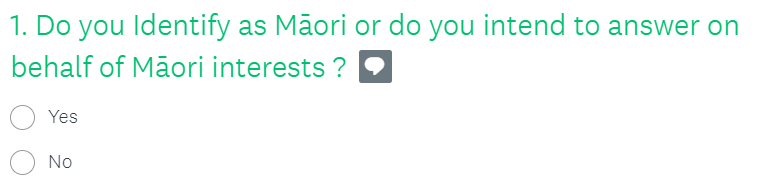


**Different forest users may have different values or views on the importance of different soil ecosystem services. As such, we need to know what best describes your relationship with planted forests in New Zealand.**


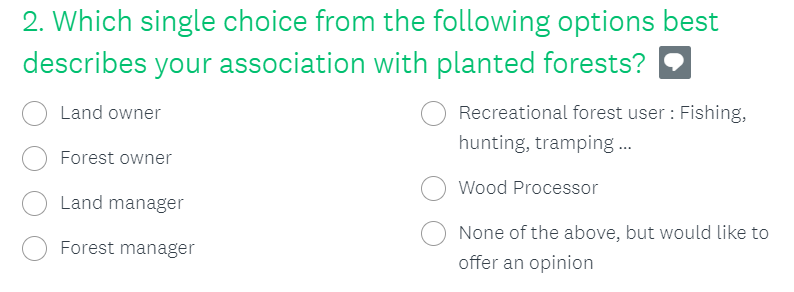


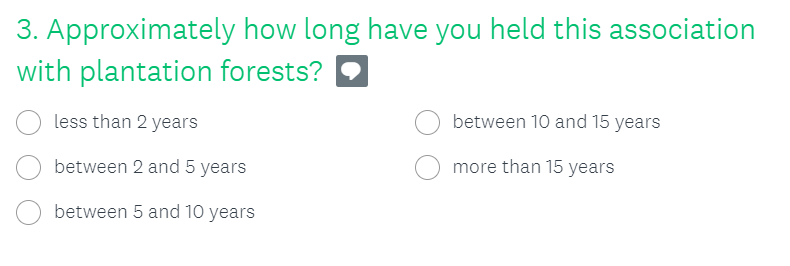


**The operations of land and forest managers/owners vary from small family businesses to large multinational organisations. Again, this may affect values and perspectives on soil biological health and ecosystem services.**


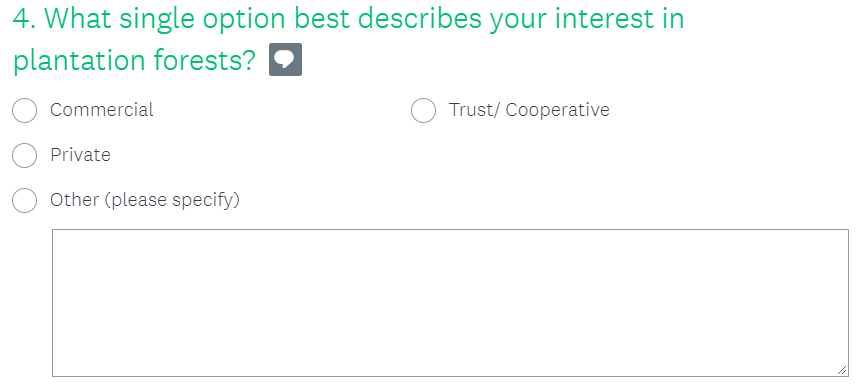


**There are a wide range of ecosystem services that soils provide. For many of these, the biology in soil has a significant role. We would like to know the importance you place on the 10 ecosystem services below.**


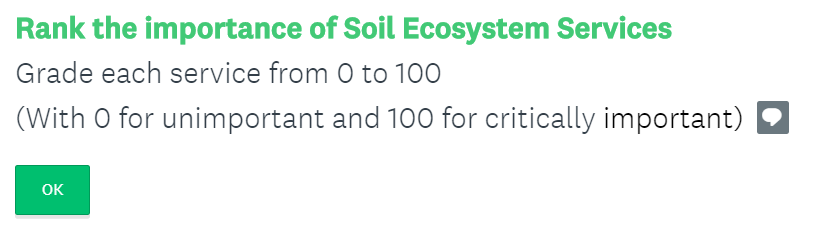


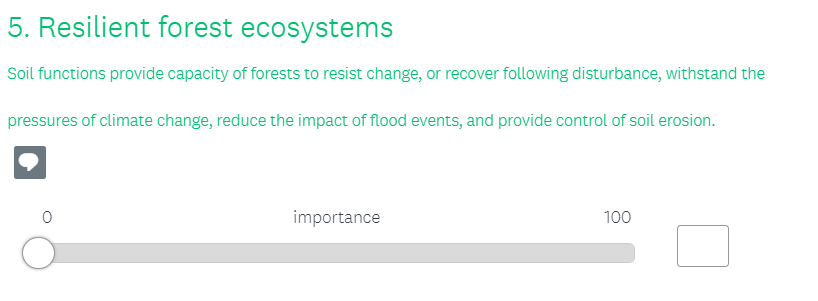


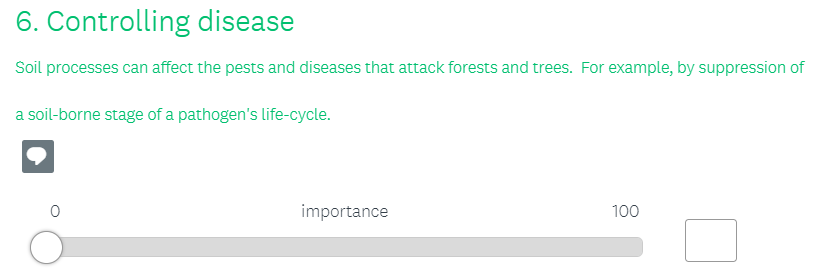


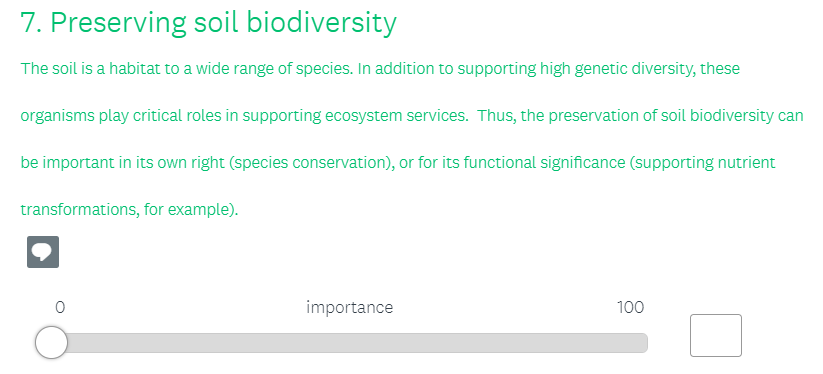


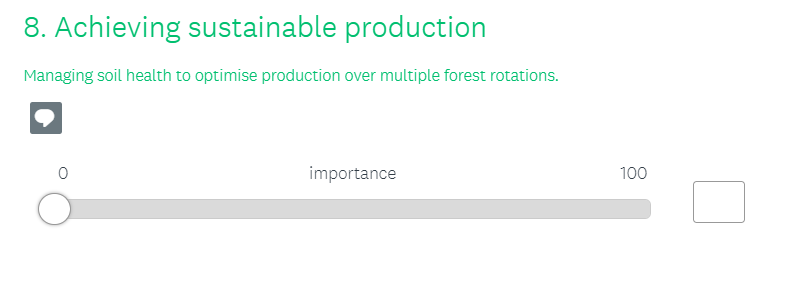


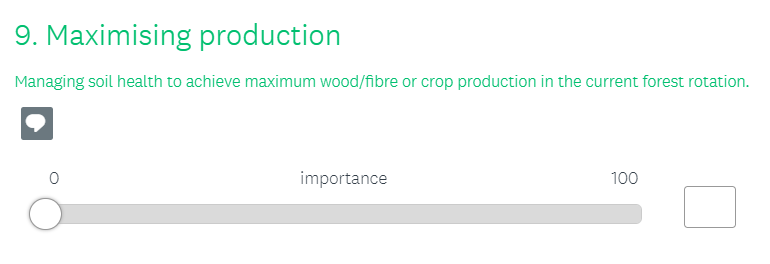


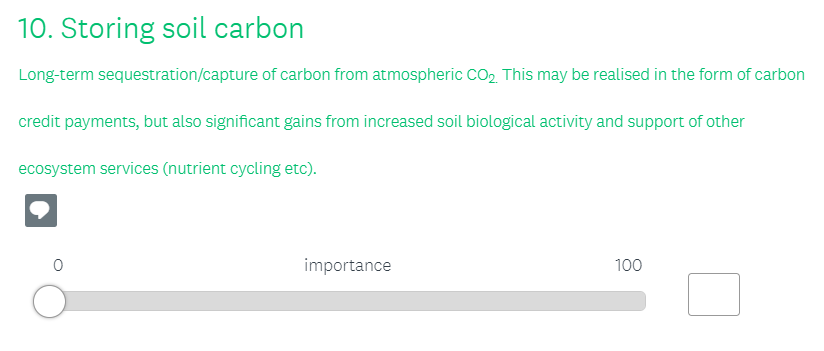


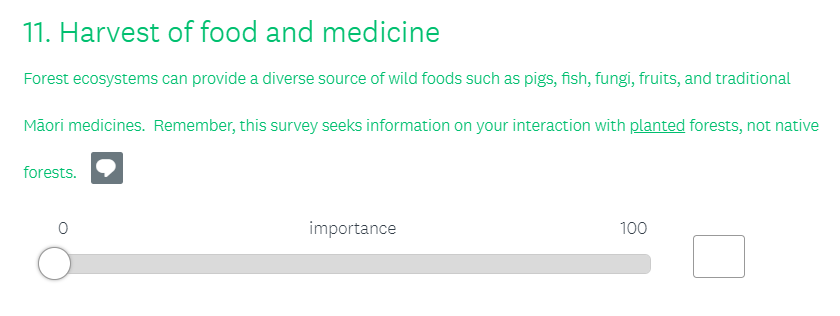


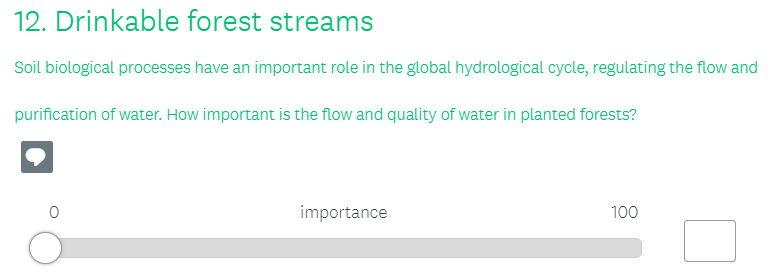


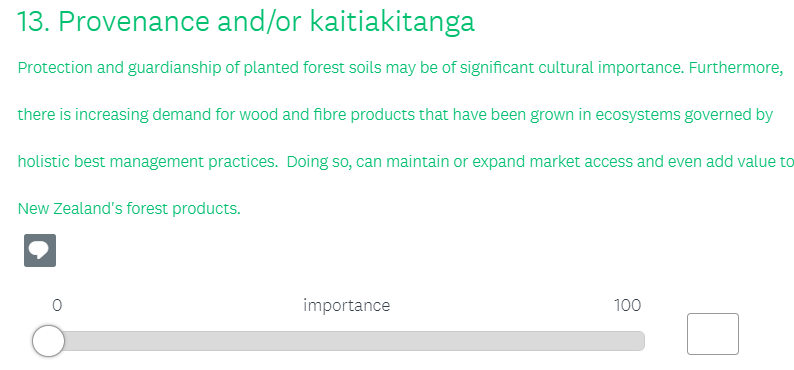


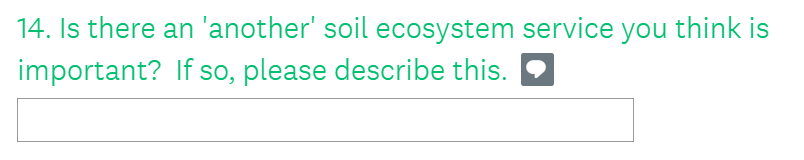


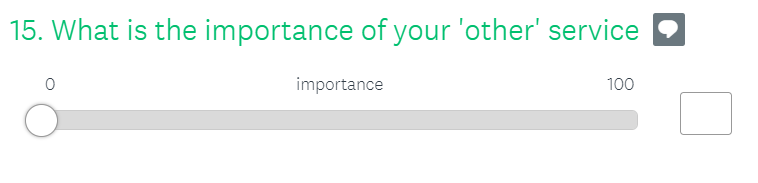


**Biological functions**

**Please consider these examples of biological functions for the next question:**

- **Maximising production**
- **Achieving sustainable production**
- **Storing soil carbon**
- **Preserving soil biodiversity**
- **Controlling pests and diseases**
- **Resilient forest ecosystems**
- **Harvest of food and medicine**
- **Drinkable forest streams**
- **Provenance & kaitiakitanga**
- **Other (please specify)**


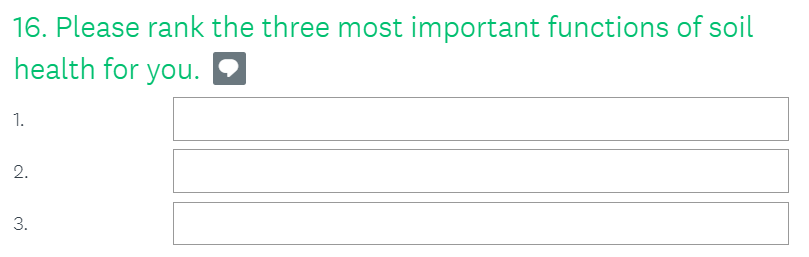


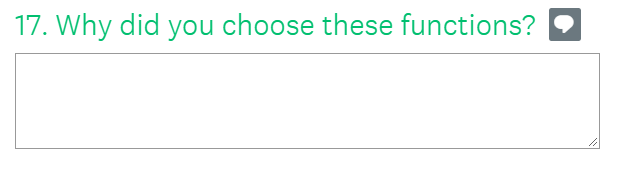


**Biodiversity in soil**

**'Biodiversity' can mean different things to different people.**

**What aspects of biodiversity are of most importance to you?**


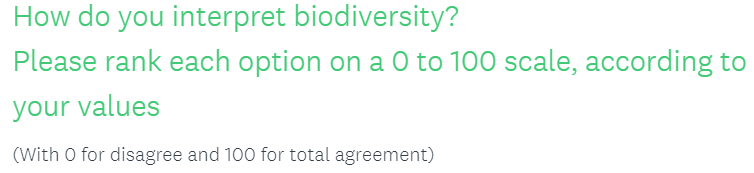


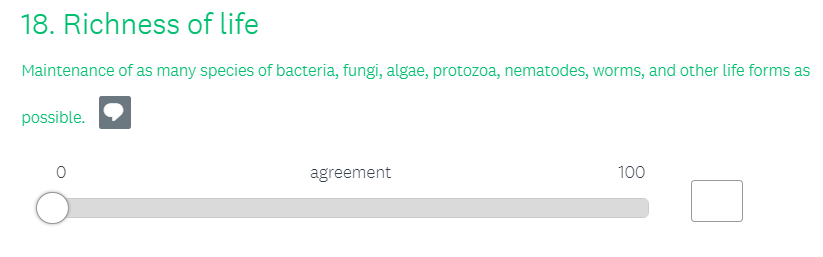


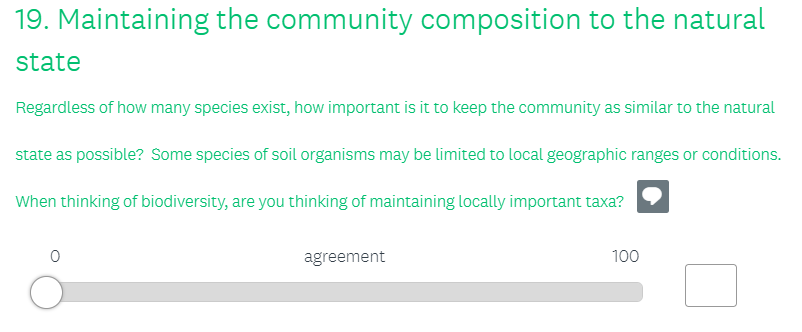


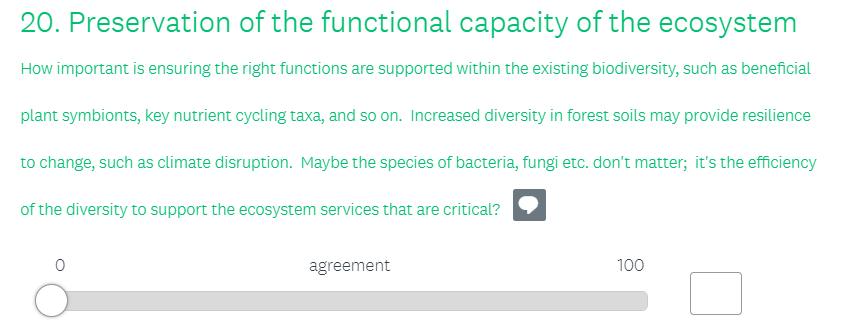


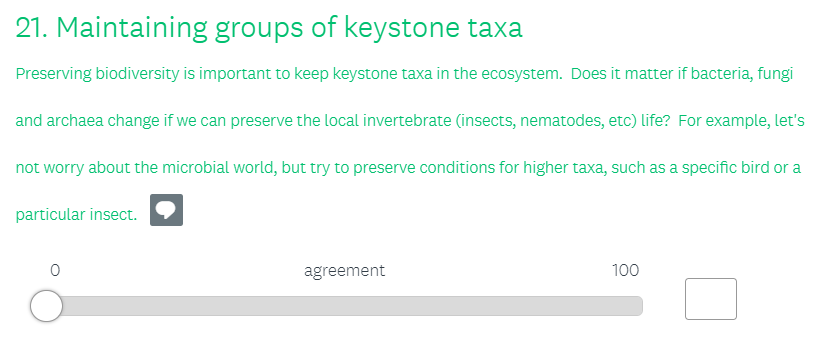


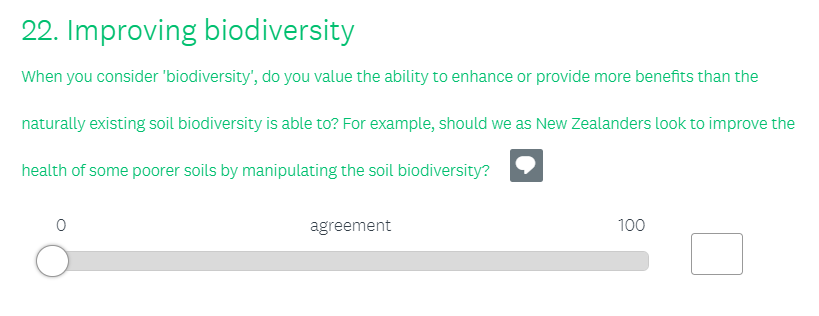


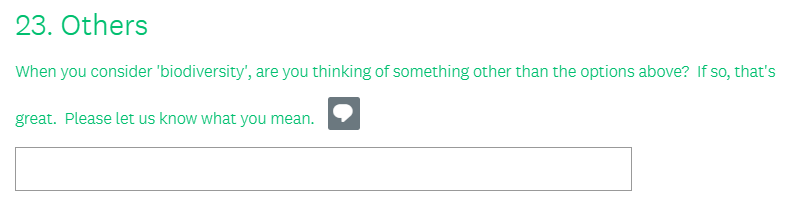


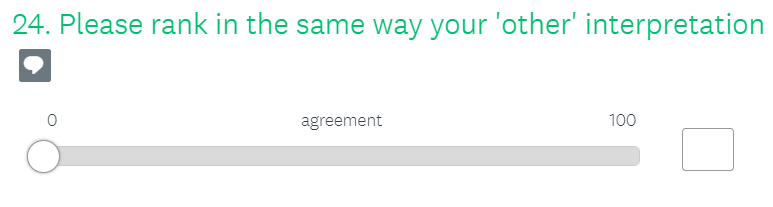


**Information on how you might currently monitor soil health and quality**

**Some forest owners/users may already sample forest soils for a variety of purposes. Are you one of these?**


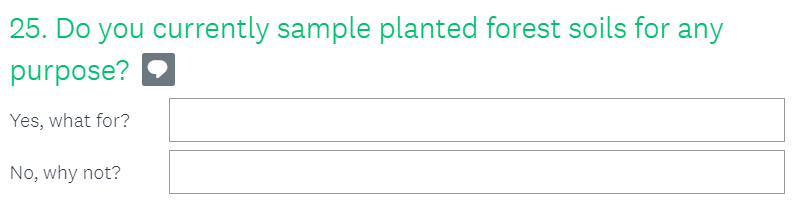


**Where in New Zealand?**

**Planted forests are present across New Zealand. Which regions best describes where you mainly interact with planted forests.**


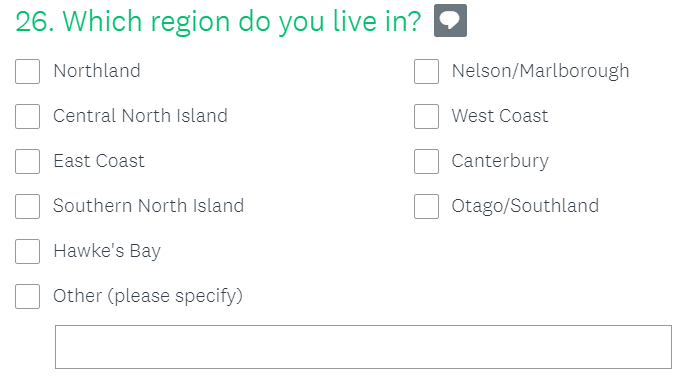

Supplement: S1 Appendix — List of questions and explanatory material used for surveying stakeholders for their valuation of forest soil ecosystem services. (DOCX) [file pone.0221291.s001.docx]
