# Supplementary material for: Stakeholder valuation of soil ecosystem services from New Zealand’s planted forests
Source: PLoS One. 2019 Aug 22;14(8):e0221291. doi: 10.1371/journal.pone.0221291 (PMC6705829; doi:10.1371/journal.pone.0221291)
Supplement: S2 Appendix — Tables of data of responses (counts and/or values) to questions posed in the survey of forest stakeholders. (DOCX) [file pone.0221291.s002.docx]

**Response to question “Which enterprise do you most strongly associate with?”**

| **Enterprise** | **Count** |
| --- | --- |
| Forest manager | 25 |
| Forest owner | 28 |
| Land manager | 6 |
| Land owner | 39 |
| Recreational forest user | 26 |
| Wood Processor | 11 |
| Other | 10 |

**Response to question “What are your region(s) of forestry interest in New Zealand?”**

| **Region** | **Count** |
| --- | --- |
| Northland | 12 |
| Central North Island | 37 |
| East Coast | 10 |
| Southern North Island | 17 |
| Hawke's Bay | 6 |
| Nelson/Marlborough | 10 |
| West Coast | 2 |
| Canterbury | 28 |
| Otago/Southland | 18 |
| Other | 12 |
| No response to question | 41 |

**Response to question “How many years of forestry related experience do you have?”**

| **Years** | **Count** |
| --- | --- |
| 0-2 | 6 |
| 2-5 | 15 |
| 5-10 | 10 |
| 10-15 | 14 |
| 15- | 100 |

**Summary of instances (counts) where respondents were asked “what are the three highest values they place on delivery of different ecosystem services?”. For instance, only 1 person ranked “Controlling pests and diseases” the highest ranked (value 3) ecosystem service.**

| **Ecosystem Service** | **Ranked top-most important – value of 3** | **Ranked second-most important – value of 2** | **Ranked third-most important – value of 1** |
| --- | --- | --- | --- |
| Controlling pests and diseases | 1 | 9 | 15 |
| Drinkable Forest Streams | 17 | 25 | 22 |
| Harvest of food and medicine | 1 | 3 | 3 |
| Achieving sustainable production | 57 | 27 | 20 |
| Maximising production | 21 | 15 | 9 |
| Preserving soil biodiversity | 8 | 13 | 20 |
| Provenance and Kaitiakitanga | 4 | 1 | 7 |
| Resilient forest ecosystems | 17 | 33 | 26 |
| Storing soil carbon | 6 | 7 | 13 |
| Other | 8 | 8 | 6 |

**Summary of instances (counts) where respondents were asked “what are the three highest values they place on delivery of different ecosystem services?” when split across Māori and non- Māori groups.**

| **Respondents identifying as Māori (n=14)** | **Ranked top-most important – value of 3** | **Ranked second-most important – value of 2** | **Ranked third-most important – value of 1** |
| --- | --- | --- | --- |
| Achieving sustainable production | 6 | 0 | 0 |
| Resilient forest ecosystems | 0 | 2 | 2 |
| Drinkable Forest Streams | 2 | 4 | 5 |
| Maximising production | 1 | 0 | 0 |
| Preserving soil biodiversity | 0 | 0 | 1 |
| Storing soil carbon | 0 | 0 | 0 |
| Controlling pests and diseases | 1 | 1 | 1 |
| Provenance and Kaitiakitanga | 1 | 3 | 1 |
| Harvest of food and medicine | 1 | 1 | 2 |
|  |  |  |  |
| **Respondents identifying as non-Māori (n=131)** | **Ranked top-most important – value of 3** | **Ranked second-most important – value of 2** | **Ranked third-most important – value of 1** |
| Achieving sustainable production | 52 | 20 | 26 |
| Resilient forest ecosystems | 18 | 26 | 31 |
| Drinkable Forest Streams | 16 | 18 | 20 |
| Maximising production | 20 | 9 | 15 |
| Preserving soil biodiversity | 9 | 20 | 15 |
| Storing soil carbon | 6 | 13 | 7 |
| Controlling pests and diseases | 1 | 14 | 8 |
| Provenance and Kaitiakitanga | 3 | 4 | 0 |
| Harvest of food and medicine | 2 | 2 | 1 |

**Summary of instances (counts) where respondents were asked “what are the three highest values they place on delivery of different ecosystem services?” when split across different user groups.**

|  | **Achieving sustainable production** | **Resilient forest ecosystems** | **Drinkable Forest Streams** | **Maximising production** | **Preserving soil biodiversity** | **Storing soil carbon** | **Controlling pests and diseases** | **Provenance and Kaitiakitanga** | **Harvest of food and medicine** |
| --- | --- | --- | --- | --- | --- | --- | --- | --- | --- |
| **Forest manager** | | | | | | | | | |
| most important – value of 3 | 11 | 6 | 0 | 3 | 1 | 1 | 1 | 0 | 0 |
| second- important – value of 2 | 4 | 4 | 3 | 9 | 1 | 0 | 2 | 0 | 0 |
| third important – value of 1 | 3 | 7 | 5 | 1 | 2 | 1 | 2 | 2 | 0 |
| **Forest owner** | | | | | | | | | |
| most important – value of 3 | 12 | 4 | 2 | 8 | 1 | 1 | 0 | 0 | 0 |
| second- important – value of 2 | 7 | 5 | 3 | 1 | 6 | 2 | 3 | 0 | 0 |
| third important – value of 1 | 2 | 8 | 3 | 3 | 6 | 3 | 2 | 1 | 0 |
| **Land manager** | | | | | | | | | |
| most important – value of 3 | 2 | 2 | 1 | 1 | 0 | 0 | 0 | 0 | 0 |
| second- important – value of 2 | 1 | 0 | 2 | 0 | 0 | 1 | 2 | 0 | 0 |
| third important – value of 1 | 2 | 1 | 0 | 0 | 0 | 2 | 0 | 0 | 0 |
| Land owner | | | | | | | | | |
| most important – value of 3 | 15 | 3 | 6 | 6 | 5 | 1 | 0 | 2 | 0 |
| second- important – value of 2 | 8 | 11 | 6 | 1 | 5 | 2 | 2 | 1 | 2 |
| third important – value of 1 | 5 | 4 | 6 | 3 | 8 | 3 | 6 | 2 | 0 |
| Recreational forest user | | | | | | | | | |
| most important – value of 3 | 8 | 1 | 5 | 0 | 2 | 2 | 1 | 2 | 3 |
| second- important – value of 2 | 0 | 7 | 9 | 2 | 4 | 0 | 0 | 0 | 1 |
| third important – value of 1 | 5 | 5 | 3 | 0 | 3 | 0 | 3 | 2 | 3 |
| **Wood processor** | | | | | | | | | |
| most important – value of 3 | 4 | 1 | 3 | 2 | 0 | 0 | 0 | 0 | 0 |
| second- important – value of 2 | 3 | 4 | 1 | 0 | 0 | 1 | 0 | 0 | 0 |
| third important – value of 1 | 2 | 1 | 2 | 1 | 1 | 1 | 2 | 0 | 0 |
| **None of the above** | | | | | | | | | |
| most important – value of 3 | 6 | 1 | 1 | 1 | 0 | 1 | 0 | 0 | 0 |
| second- important – value of 2 | 3 | 2 | 1 | 2 | 0 | 1 | 0 | 0 | 0 |
| third important – value of 1 | 1 | 2 | 3 | 1 | 0 | 3 | 0 | 0 | 0 |
