# Supplementary material for: Stakeholder valuation of soil ecosystem services from New Zealand’s planted forests
Source: PLoS One. 2019 Aug 22;14(8):e0221291. doi: 10.1371/journal.pone.0221291 (PMC6705829; doi:10.1371/journal.pone.0221291)
Supplement: S1 Table — Summary SIMPER testing of variables contributing towards the differences between Māori and non-Māori stakeholder quantification of forest soil ecosystem services. Average dissimilarity = 15.91. (DOCX) [file pone.0221291.s003.docx]

| **Variable** | **Average value (%)^a^** | | **Average dissimilarity^b^** | **Contribution to separation (%)^c^** |
| --- | --- | --- | --- | --- |
|  | **Non-Māori** | **Māori** |  |  |
| **Achieving sustainable harvest** | **49.5** | **86.1** | **2.93** | **18.4** |
| **Provenance and kaitiakitanga** | **58.3** | **95.1** | **2.76** | **17.4** |
| **Maximising production** | **77.1** | **77.5** | **1.92** | **12.0** |
| **Storing soil carbon** | **75.7** | **80.7** | **1.71** | **10.7** |
| **Controlling pests and diseases** | **76.1** | **87.4** | **1.67** | **10.5** |
| **Preserving soil biodiversity** | **80.7** | **84.3** | **1.45** | **9.12** |

**^a^ Average percentage ranking of each soil ecosystem system service by Māori and non-Māori stakeholder**

**^b^ Average Bray-Curtis similarity between all pairs of Māori and non-Māori stakeholder ecosystem services**

**^c^ Percentage contribution to the overall average dissimilarity (15.91%) in ecosystem services by Māori and non-Māori stakeholders**
