# Supplementary figures and images for: Stakeholder valuation of soil ecosystem services from New Zealand’s planted forests
Source: PLoS One. 2019 Aug 22;14(8):e0221291. doi: 10.1371/journal.pone.0221291 (PMC6705829; doi:10.1371/journal.pone.0221291)

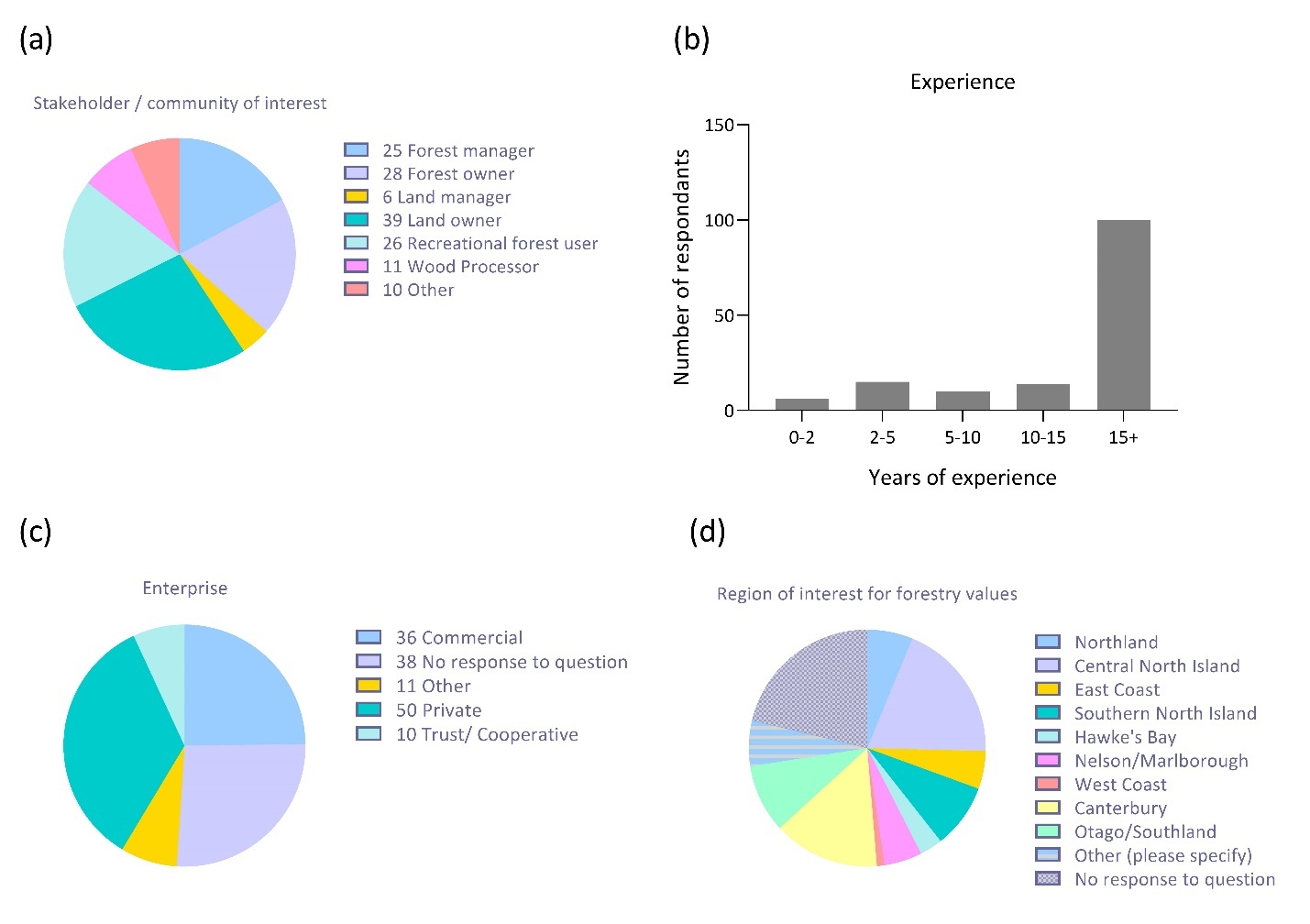

Supplement: S1 Fig — Breakdown of survey respondents by (a) sector/community of interest, (b) years of experience, (c) type of enterprise (private v commercial operator etc), and (d) the region of forestry interest. Note for ‘region of interest’ that more than one option was available for the survey respondents. (DOCX) [file pone.0221291.s004.docx]

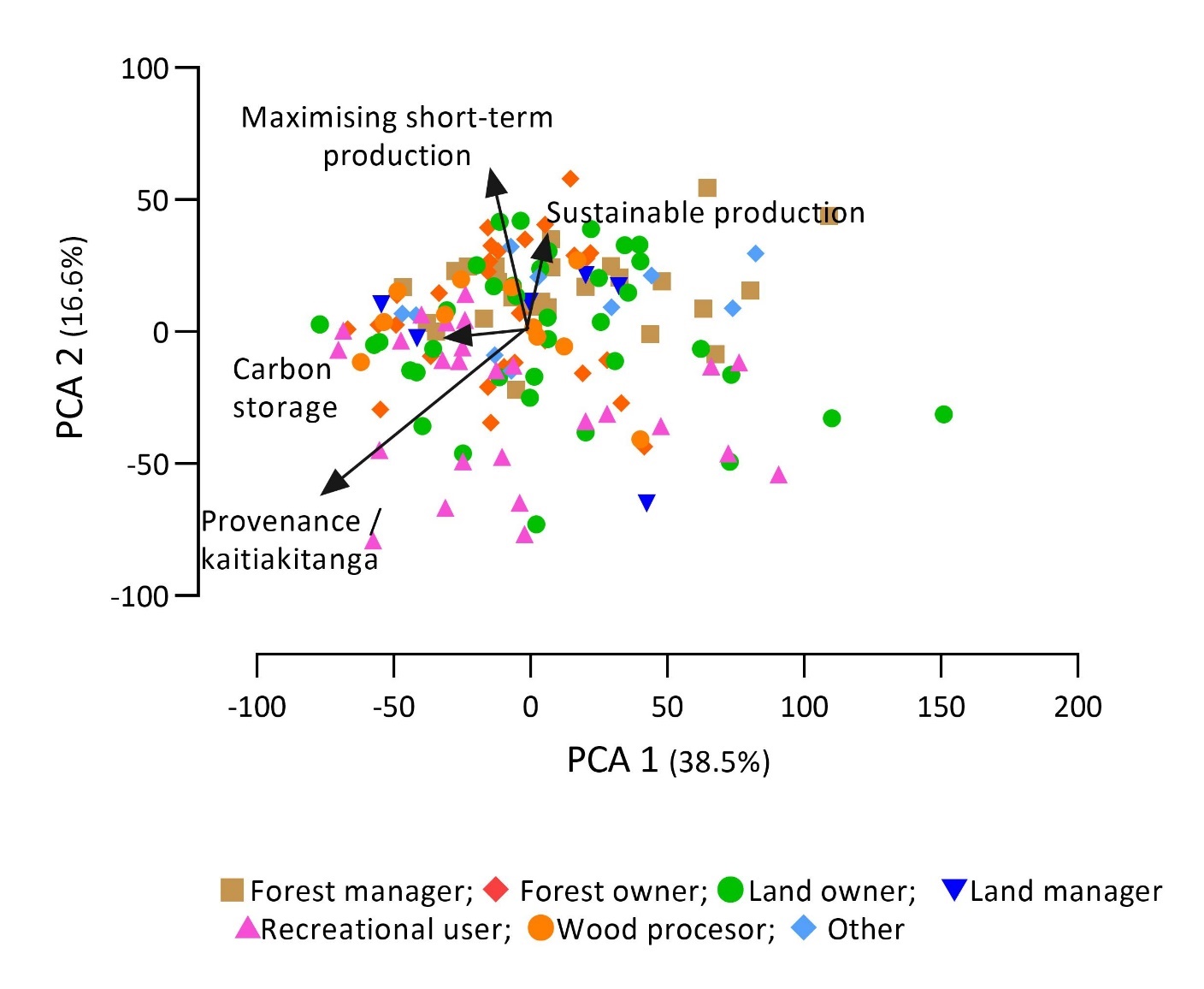

Supplement: S2 Fig — Principle components analysis (PCA) ordination plot showing separation in forest stakeholders’ valuation of overall forest soil ecosystem services. Lines indicate the ecosystem values (variables) associated with separation in responses in each direction; the length of the lines is proportional to their strength. Variables were selected based on ranking in SIMPER analysis conducted between pairs of stakeholder groups found to significantly differ (p<0.05). (DOCX) [file pone.0221291.s005.docx]

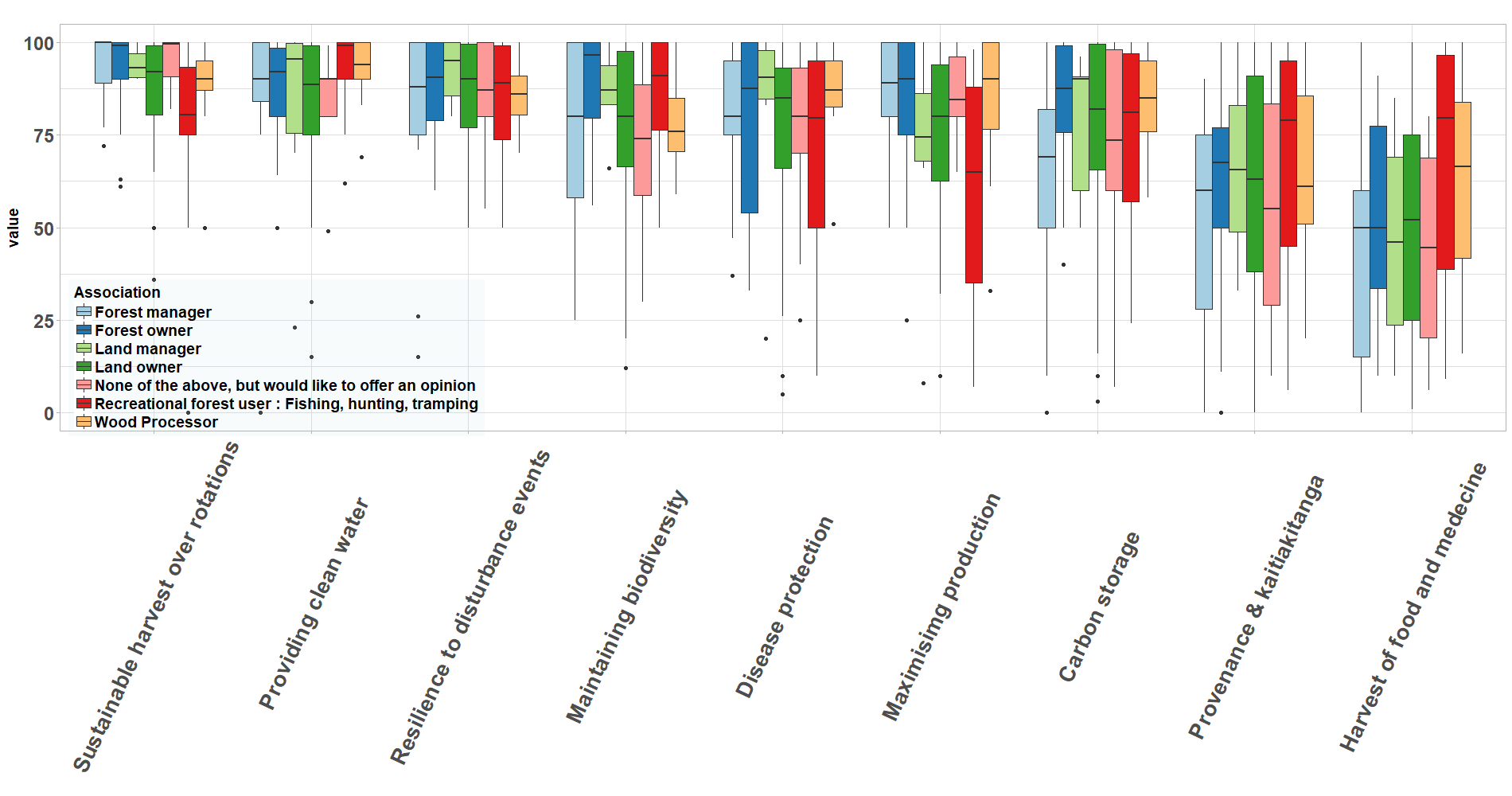

Supplement: S4 Fig — Values placed by different stakeholders (x-axis) on a range of planted forest ecosystem services. Note many were ‘valued’ at or near 100, giving a skewed perspective of the distributions. Data includes responses from both Māori and non-Māori communities. Maintaining biodiversity refers to soil biodiversity only. (DOCX) [file pone.0221291.s007.docx]

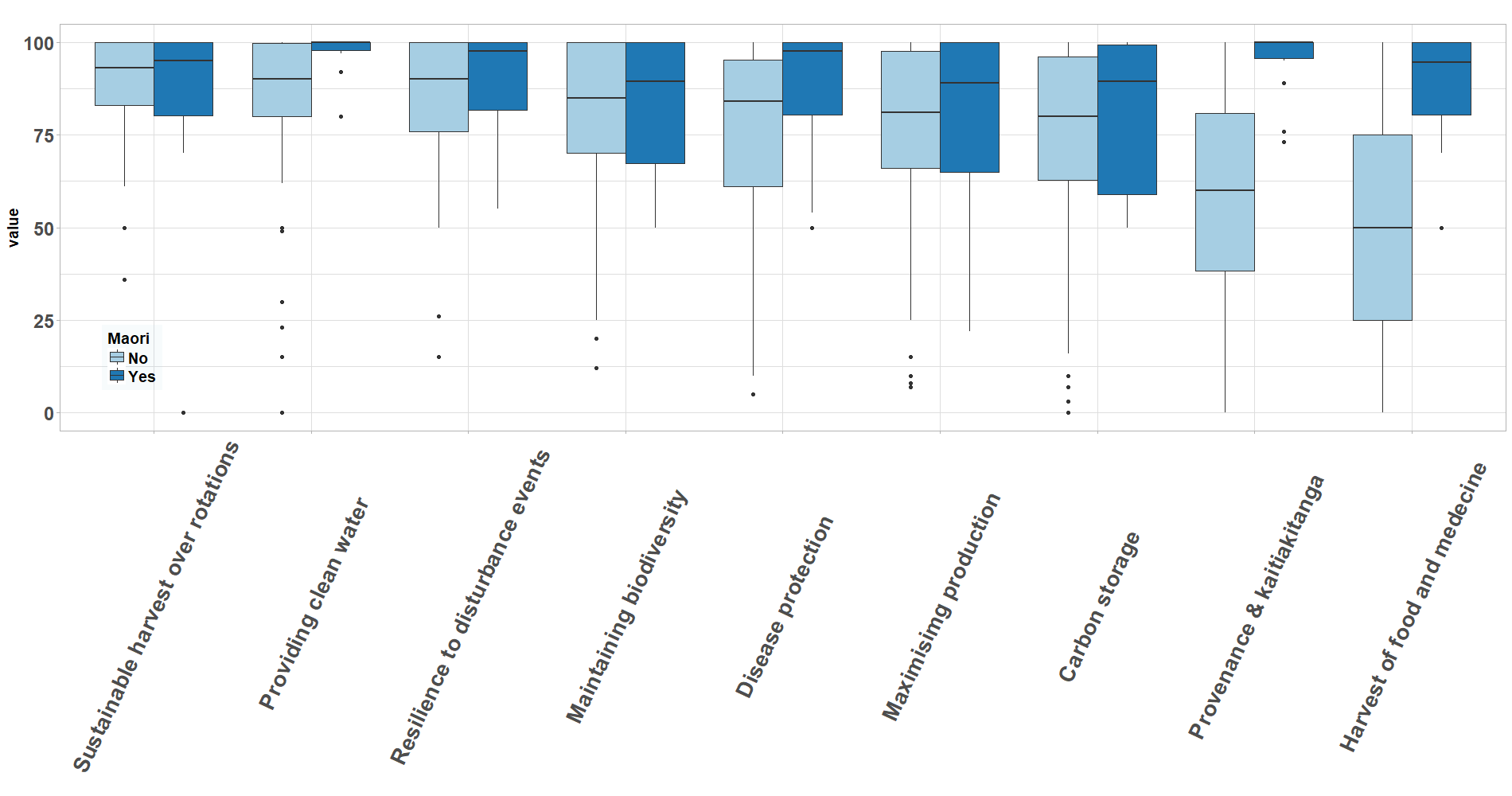

Supplement: S5 Fig — Values (Y-axis) placed by survey respondents identifying as Māori and non-Māori (x-axis) on a range of planted forest ecosystem services. Note many ‘valued’ at or near 100 (Y-axis), giving a skewed perspective of the distributions. Data includes responses from across all stakeholders. Maintaining biodiversity refers to soil biodiversity only. (DOCX) [file pone.0221291.s008.docx]
