# Supplementary material for: Stakeholder valuation of soil ecosystem services from New Zealand’s planted forests
Source: PLoS One. 2019 Aug 22;14(8):e0221291. doi: 10.1371/journal.pone.0221291 (PMC6705829; doi:10.1371/journal.pone.0221291)
Supplement: Supplementary file 5 [file pone.0221291.s006.docx]

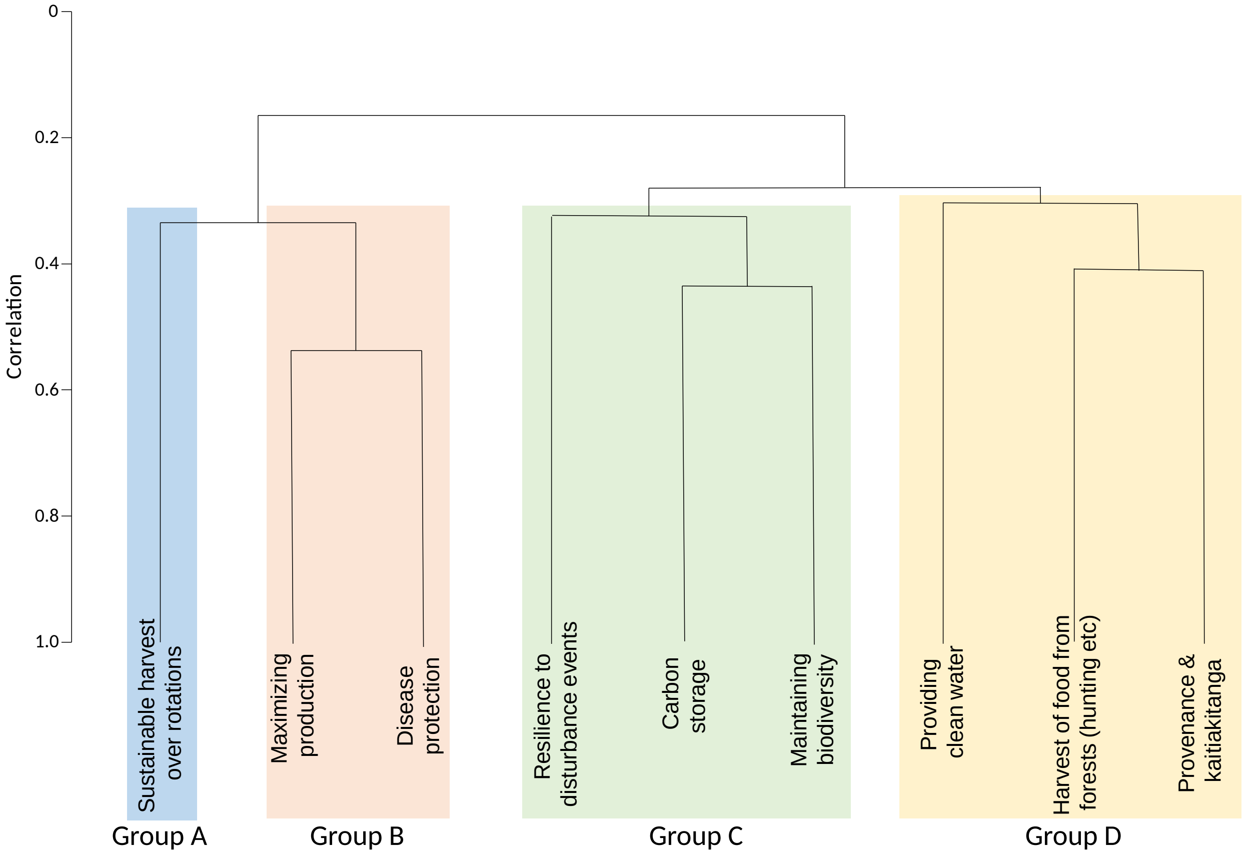
0
0.2
0.4
0.6
0.8
1.0
Correlation
Sustainable harvest over rotations
Maximizing production
Disease protection
Resilience to disturbance events
Carbon storage
Maintaining biodiversity
Providing clean water
Harvest of food from forests (hunting etc)
Provenance & kaitiakitanga
Group A
Group B
Group C
Group D
